# Resveratrol Improves Tube Formation in AGE-Induced Late Endothelial Progenitor Cells by Suppressing Syndecan-4 Shedding

**DOI:** 10.1155/2018/9045976

**Published:** 2018-04-10

**Authors:** Han Wu, Zheng Chen, Jian-Zhou Chen, Jun Xie, Biao Xu

**Affiliations:** Department of Cardiology, Drum Tower Hospital, Nanjing University Medical School, Nanjing 210008, China

## Abstract

Dysfunction of endothelial progenitor cells (EPCs) contributes to cardiovascular complications in diabetes, and resveratrol has been shown to improve EPC functions. Syndecan-4 (Synd4), a cell surface heparin sulfate proteoglycan, has been shown to promote neovascularization. Thus, the present study was performed to determine whether resveratrol promoted angiogenesis of EPCs by regulating Synd4. Late EPCs were isolated from human peripheral blood and stimulated with AGEs. Western blot showed that AGEs induced Synd4 shedding in a dose- and time-dependent manner. AGE-induced Synd4 shedding was partly reversed by NAC or resveratrol, along with normalized ROS production. Overexpression of Synd4 or pretreatment of resveratrol reversed AGE-impaired tube formation of EPCs and regulated the Akt/eNOS pathway. Furthermore, resveratrol suppressed Synd4 shedding via the inhibition of oxidative stress and improved tube formation of late EPCs via the regulation of the Synd4/Akt/eNOS pathway.

## 1. Introduction

Cardiovascular complications represent the principal course of morbidity and mortality in diabetes mellitus (DM) [1]. Several studies have demonstrated that an impaired endothelial function was a critical factor for the development of diabetic cardiovascular complications [2–4]. Endothelial progenitor cells (EPCs) are a subset of bone-marrow-derived cells contributing to neovascularization and reendothelialization in various diseases [5]. EPCs contain two types of cells (early EPCs and late EPCs). Early EPCs are generated in 7 days and exhibit high-cytokine release. The long-term culture of “early EPCs” yielded colony-forming units with a more mature endothelial cell phenotype and a capacity to form vascular networks, which were named “late EPCs” [6]. Several studies have indicated impaired tube formation of EPCs in diabetes [7, 8], however, the underlying mechanisms remain unclear.

Syndecan-4 (Synd4), one member of the syndecan family, is a transmembrane heparin sulfate proteoglycan (HSPG) in various cells. There is widespread agreement that Synd4 mediates many signaling pathways as a coreceptor for some growth factors [9]. However, extracellular domains of Synd4 (ext-Synd4) may be released into the serum via proteolytic cleavage under some conditions including diabetes, which is a process known as shedding [10, 11]. In recent years, Synd4 has become increasingly attractive as a critical regulator in angiogenesis [11, 12]. However, few attempts have been made to investigate the role of Synd4 in the angiogenesis of EPCs and there are few effective methods to prevent Synd4 shedding.

Resveratrol, an antioxidant, has been shown to ameliorate oxidative stress-induced complications associated with diabetes [13]. Several studies have demonstrated that resveratrol improved EPC functions in vitro [14, 15]. However, the underlying mechanisms have yet to be elucidated. Here, the present study was carried out to explore the effect of resveratrol on Synd4 shedding and tube formation in AGE-induced EPCs.

## 2. Methods

### 2.1. Isolation and Characterization of Late EPCs

**Figure 1:**
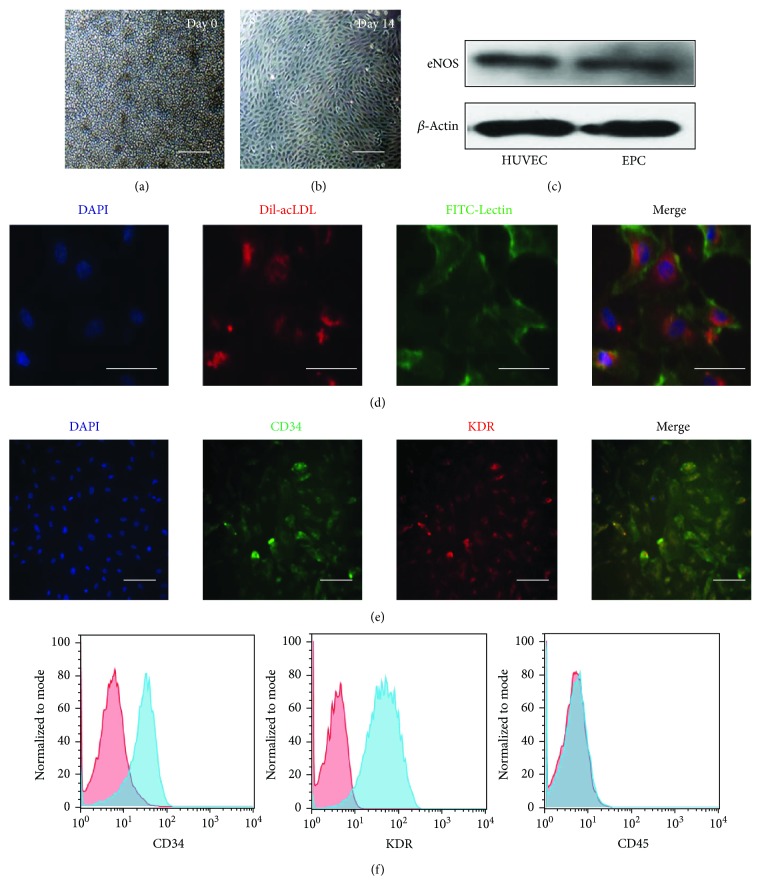
Characterization of late EPCs by a light microscope, Western blot, fluorescence microscope, and flow cytometry. (a) MNCs were isolated and plated on a culture dish on the first day. Bar = 100 *μ*m. (b) Morphology of late EPCs in culture 14 days later. Bar = 100 *μ*m. (c) Expressions of eNOS in HUVECs and late EPCs were detected by Western blot, and HUVECs were used as positive controls. (d) Cultured late EPCs stained with DAPI (blue), Dil-acLDL (red), and FITC-Lectin (green). Bar = 10 *μ*m. (e) Late EPCs were identified by representative markers including CD34 and KDR. Bar = 25 *μ*m. (f) Flow cytometric analysis of late EPCs after immunolabelling with CD34, KDR, and CD45.

All experiments were performed following an institutionally approved protocol in accordance with the Medical Ethics Committee of Drum Tower Hospital affiliated to Nanjing University Medical School. Late EPCs were isolated from healthy volunteers as previously described [16]. Briefly, mononuclear cells were isolated from peripheral blood by density gradient centrifugation. Then, the cells (Figure 1(a)) were supplemented with endothelial cell growth medium-2 (EGM-2) (Lonza) composed of endothelial cell basal medium-2 (EBM-2), 5% fetal bovine serum, and growth factors. The medium was changed every 3 days during the period. Two to 3 weeks later, late EPCs appeared and showed cobblestone-like morphology (Figure 1(b)). Western blot showed eNOS expressions in late EPCs as well as in endothelial cells (Figure 1(c)). The cells were characterized by the uptake of a 1,1-dioctadecyl-3,3,3,3-tetramethylindocarbocyanine-labeled low-density lipoprotein (Dil-acLDL; Molecular Probes) and by binding to fluorescein-isothiocyanate-conjugated lectin (FITC-Lectin; Sigma-Aldrich) as previously described [16] (Figure 1(d)). In addition, the cells were detected by immunocytochemistry using FITC-CD34 (progenitor cell marker, BD Biosciences) and allophycocyanin-KDR (endothelial marker, APC-KDR, R&D Systems) and DAPI was used to stain nuclei (Figure 1(e)). Furthermore, flow cytometry was used to determine the expression of the progenitor cell marker CD34 (BD Biosciences), endothelial marker KDR (Caltag Laboratories), and leukocyte marker CD45 (R&D Systems). As shown in Figure 1(f), late EPCs were positive for CD34 and KDR, and negative for CD45.

### 2.2. Western Blot

Cell protein was extracted in a cell lysis buffer in the presence of a 0.1% protease inhibitor (Sigma-Aldrich). Protein concentration was detected by a BCA protein assay kit (Pierce) according to the manufacturer's instructions. After being mixed with a loading buffer in boiling water, equal proteins were separated through SDS-PAGE and transferred to PVDF membranes. Then, the membranes were blocked with 10% milk and incubated with primary antibodies at 4°C overnight including *β*-actin (1 : 2000, Santa Cruz Biotechnology), Synd4 (1 : 500, LifeSpan BioSciences), pAkt (1 : 1000, Cell Signaling Technology), Akt (1 : 1000, Cell Signaling Technology), peNOS (1 : 1000, Cell Signaling Technology), and eNOS (1 : 1000, Cell Signaling Technology). After being washed with PBST, the membranes were incubated with an appropriate secondary antibody conjugated to HRP (1 : 1000, Bioworld). The reactions were detected by chemiluminescence reagents and images were gained by exposure to films.

### 2.3. Measurement of ROS Production

Here we measured intracellular ROS production in late EPCs using the fluorescent probe 2′-7′-dichlorofluorescin diacetate (DCFH-DA; Sigma-Aldrich). After the medium was removed, late EPCs were incubated with 5 *μ*mol/l of the DCFH-DA probe in serum-free media at 37°C for 30 min. The level of intracellular ROS genesis was examined under a fluorescence microscope.

### 2.4. Tube Formation

Matrigel was used to detect tube formation of late EPCs. The cells were added to a 96-well plate precoated with Matrigel (BD Biosciences). 12 hours later, the enclosed networks of tubes from six random high-power microscope fields were examined under a microscope.

### 2.5. Recombinant Adenoviral Infection of EPCs

Construction and preparation of recombinant adenoviruses were introduced previously [12]. When late EPCs reached approximately 70% confluence in fresh serum-free medium, they were transiently transduced with adenovirus overexpressing Synd4 (ad Synd4) or adenovirus with no Synd4 (ad null) at multiplicities of infection (MOI) of 80.

### 2.6. Statistics

Statistical analysis was conducted using SPSS software. All experiments were replicated at least three times independently. The data were represented as means ± SEM. Comparisons of results between two groups were evaluated by Student's *t*-test, multiple group comparisons were done with one-way ANOVA, and a *P* value of 0.05 was considered as the significance threshold.

## 3. Results

### 3.1. Culture and Characterization of Late EPCs from Human Peripheral Blood

Monocytes were isolated from human peripheral blood (Figure 1(a)), and the cultured cells, which were named “late EPCs”, displayed a characteristic cobblestone morphology two weeks later (Figure 1(b)). Meanwhile, Western blot showed that endothelial nitric oxide synthase (eNOS), abundantly expressed in endothelial cells, was expressed in both HUVECs and EPCs (Figure 1(c)). To identify the purity and phenotype of EPCs, the cells were characterized by immunofluorescence. Most adherent cells (>90%) took up Dil-acLDL and were labeled with FITC-Lectin (Figure 1(d)). Figure 1(e) showed that the characterized EPCs were proved by double staining with CD34 (stem cell marker) and KDR (endothelial cell lineage antigen). Furthermore, the cells were characterized using flow cytometry. Late EPCs expressed CD34 and KDR, but not the leukocyte marker CD45 (Figure 1(f)).

### 3.2. AGEs Induced Synd4 Shedding in Late EPCs

**Figure 2:**
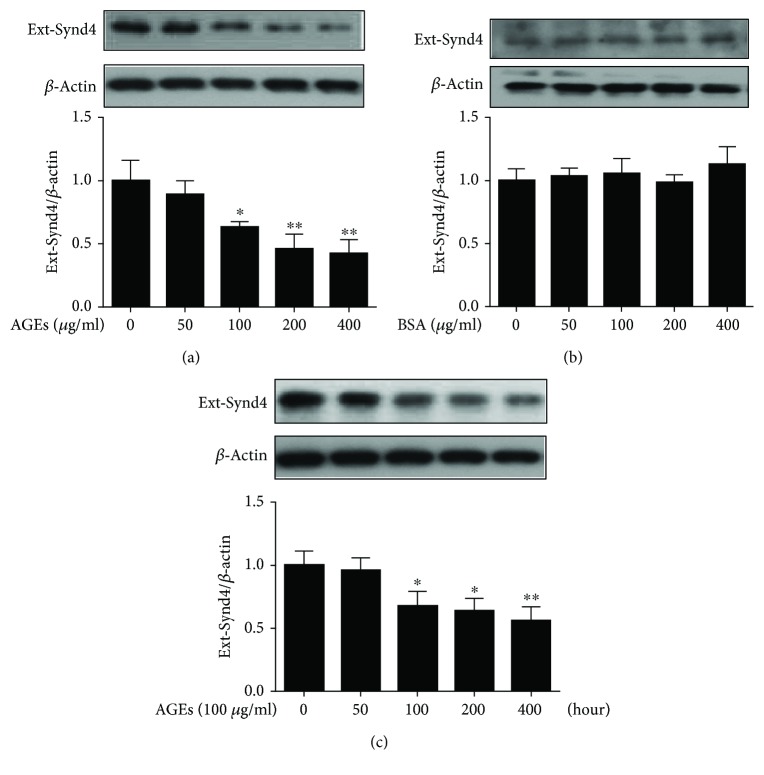
AGEs downregulated Synd4 expressions in late EPCs. (a) Effect of different AGE concentrations on the expression of Synd4 in late EPCs. (b) Effect of different BSA concentrations on the expressions of Synd4 in late EPCs. (c) Effect of 100 *μ*g/ml of AGEs on the expressions of Synd4 in late EPCs for different times. *N* = 3, ^∗^*p* < 0.05 versus the control group; ^∗∗^*p* < 0.01 versus the control group.

To investigate the role of AGEs on Synd4 shedding, EPCs were incubated with 0–400 *μ*g/ml of AGEs for 24 hours. As shown in Figure 2(a), AGEs elicited a dose-dependent decrease in Synd4 expressions. In addition, treatment with AGEs at a dose of 100 *μ*g/ml reached a significant difference. As a control, BSA had no effect on Synd4 expressions (Figure 2(b)). EPCs were then incubated with 100 *μ*g/ml of AGEs for 0–48 hours, and it was observed that treatment of EPCs with AGEs for 24 hours had a most robust effect on Synd4 shedding (Figure 2(c)). Thus, EPCs stimulated with 100 *μ*g/ml of AGEs for 24 hours were selected in subsequent experiments.

### 3.3. Resveratrol Attenuated AGE-Induced Synd4 Shedding via the Regulation of Oxidative Stress and sirt1 Expression in Late EPCs

**Figure 3:**
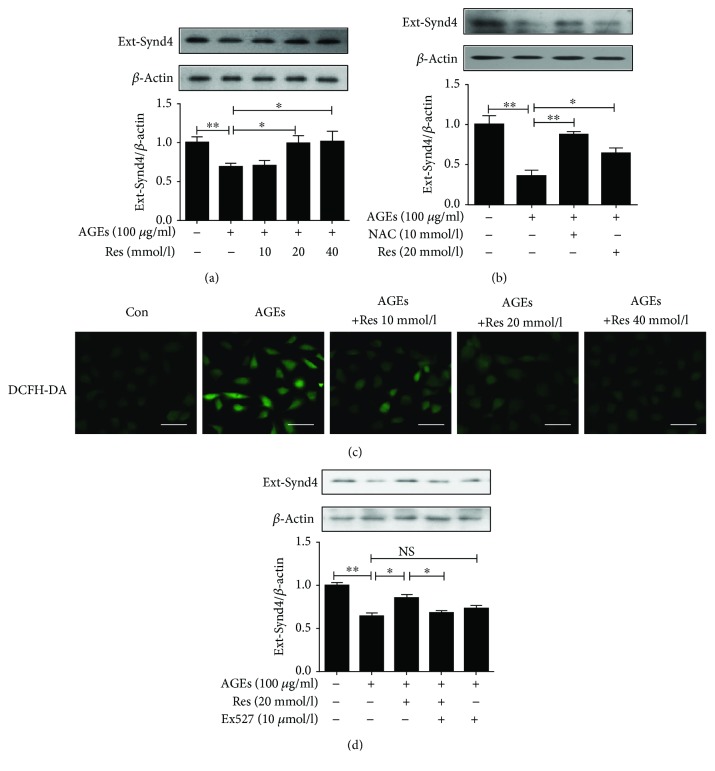
Resveratrol mimicked the effect of NAC on Synd4 shedding in AGE-induced late EPCs. (a) Resveratrol attenuated the shedding of Synd4 in AGE-induced late EPCs. (b) Both resveratrol and NAC inhibited Synd4 shedding in AGE-induced late EPCs. (c) Resveratrol attenuated ROS production in late EPCs induced by AGEs. (d) Treatment with Ex527 reversed the effect of resveratrol on AGE-induced Synd4 shedding from late EPCs. *N* = 3. NS, no significant difference between groups. ^∗^*p* < 0.05 between two groups, ^∗∗^*p* < 0.01 between two groups.

In this section, we undertook the current study to explore how resveratrol regulated AGE-induced Synd4 shedding in late EPCs. As illustrated in Figure 3(a), resveratrol treatment had a tremendous beneficial effect in terms of inhibiting Synd4 shedding in AGE-induced EPCs. Furthermore, resveratrol partially abolished Synd4 shedding induced by AGEs and attenuated AGE-induced ROS production in late EPCs (Figures 3(b) and 3(c)). AGE-mediated Synd4 shedding was attenuated in late EPCs by pretreatment of NAC, an inhibitor of ROS production (Figure 3(b)). As previous reports have shown the critical role of sirt1 in AGE-induced EPCs and resveratrol has been considered as an activator of sirt1, we asked whether resveratrol inhibited Synd4 shedding via the regulation of sirt1. The results showed that the inhibition of Synd4 shedding of resveratrol was abrogated by pretreatment with Ex527, an inhibitor of sirt1, suggesting that resveratrol mediated the amelioration of Synd4 shedding via the activation of sirt1 (Figure 3(d)).

### 3.4. Overexpression of Synd4 or Treatment with Resveratrol Reversed AGE-Induced Impaired Tube Formation of Late EPCs

**Figure 4:**
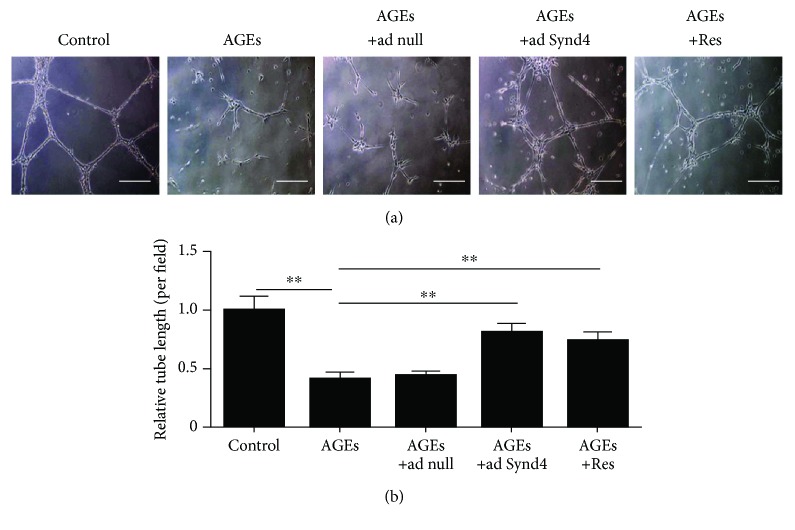
Effect of resveratrol on tube formation in AGE-induced late EPCs. (a) Light microscopic images of the tube formation in different groups. (b) Analysis of tube formation in different groups. ^∗∗^*p* < 0.01 between two groups. *n* = 5, bar = 100 *μ*m. Ad null, adenoviral constructs with no Synd4; ad Synd4, adenoviral constructs expressing human Synd4.

In this section, as depicted in Figure 4, overexpression of Synd4 improved the impaired tube formation upon stimulation of AGEs. Furthermore, the effect of Synd4 was mimicked by resveratrol treatment, suggesting that the stimulatory effect of resveratrol on capillary-like tube formation might be partly mediated by suppressing Synd4 shedding.

### 3.5. Overexpression of Synd4 Activated Akt and eNOS

**Figure 5:**
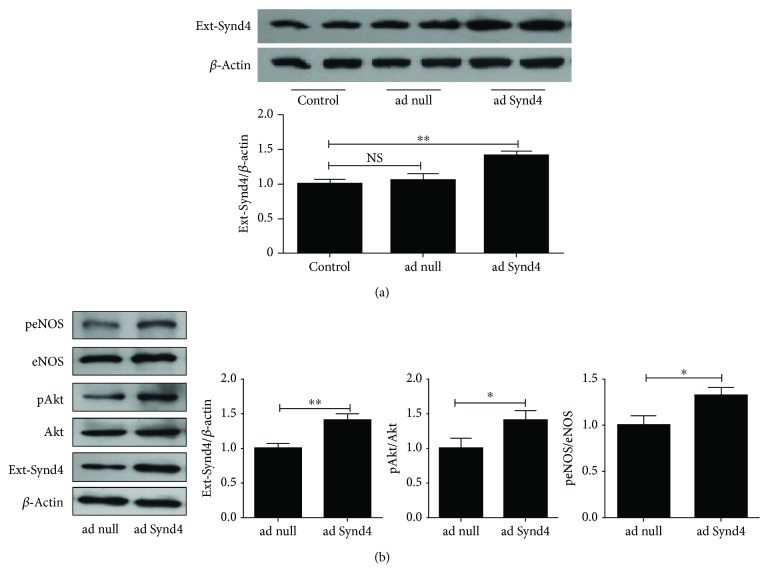
Overexpression of Synd4 promoted the activation of the Akt/eNOS pathway. (a) Western blot analysis of Synd4 in late EPCs transfected with adenovirus containing the Synd4 gene (ad Synd4) or control adenovirus (ad null). (b) Effect of Synd4 overexpression on the activation of the Akt/eNOS pathway. NS, no significant difference between groups. ^∗^*p* < 0.05 between groups, ^∗∗^*p* < 0.01 between groups.

Early reports have demonstrated the contribution of the Akt/eNOS pathway to tube formation, and Synd4 was considered to regulate Akt activation in endothelial cells, so we assessed phosphorylation of Akt and eNOS with overexpressing Synd4. As shown in Figure 5(a), late EPCs transfected with adenovirus containing the Synd4 gene (ad Synd4) displayed higher Synd4 expressions compared to control. However, it was similar between control late EPCs and those cells transfected with control adenovirus (ad null). In addition, we found an upregulation of Synd4 followed by an increased expression of pAkt/Akt and paralleled by increased activation of eNOS in late EPCs overexpressing Synd4 (Figure 5(b)).

### 3.6. Resveratrol Inhibited Inactivation of the Akt/eNOS Pathway Induced by AGEs in Late EPCs

**Figure 6:**
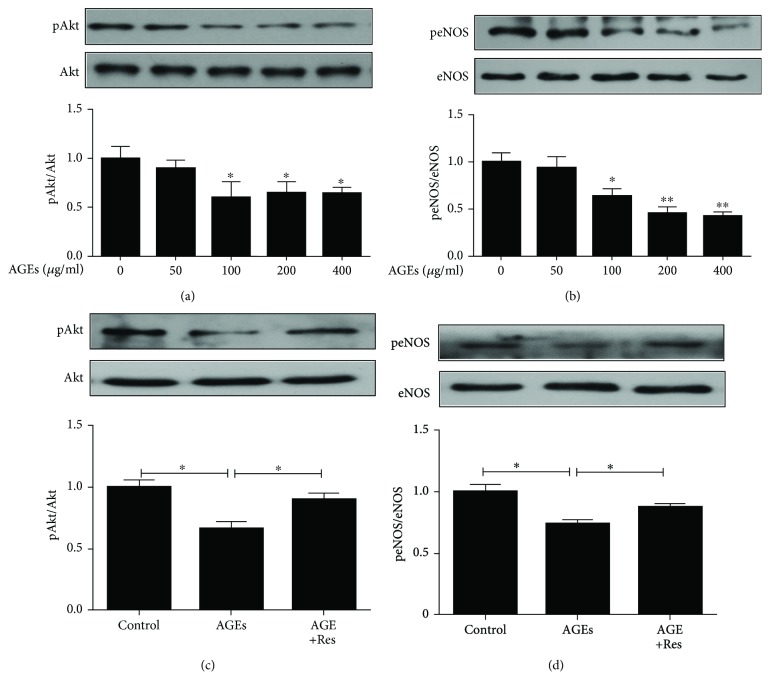
Reduced phosphorylation of Akt/eNOS induced by AGEs was reversed by treatment with resveratrol. (a) AGEs suppressed phosphorylation of Akt in a dose-dependent manner in late EPCs. *N* = 3, ^∗^*p* < 0.05 versus control. (b) AGEs suppressed phosphorylation of eNOS in a dose-dependent manner in late EPCs. *N* = 3, ^∗^*p* < 0.05 versus control, ^∗∗^*p* < 0.01 versus control. (c) Resveratrol induced activation of Akt in AGE-induced late EPCs. *N* = 3, ^∗^*p* < 0.05 between groups. (d) Resveratrol induced activation of eNOS in AGE-induced late EPCs. *N* = 3, ^∗^*p* < 0.05 between groups.

Since we had previously reported that Synd4 overexpression induced its downstream proteins, we hypothesized that resveratrol regulated the Synd4/Akt/eNOS pathway in AGE-induced late EPCs. To test this hypothesis, we first detected phosphorylation of Akt/eNOS in late EPCs incubated with different concentrations of AGEs. As depicted in Figures 6(a) and 6(b), AGEs elicited a dose-dependent decrease in phosphorylation of Akt/eNOS. However, the stimulatory effect of AGEs on late EPCs was partly abolished by pretreatment with resveratrol (Figures 6(c) and 6(d)), corroborating the notion that resveratrol regulated AGE-induced Synd4 shedding in late EPCs and its downstream proteins.

## 4. Discussion

**Figure 7:**
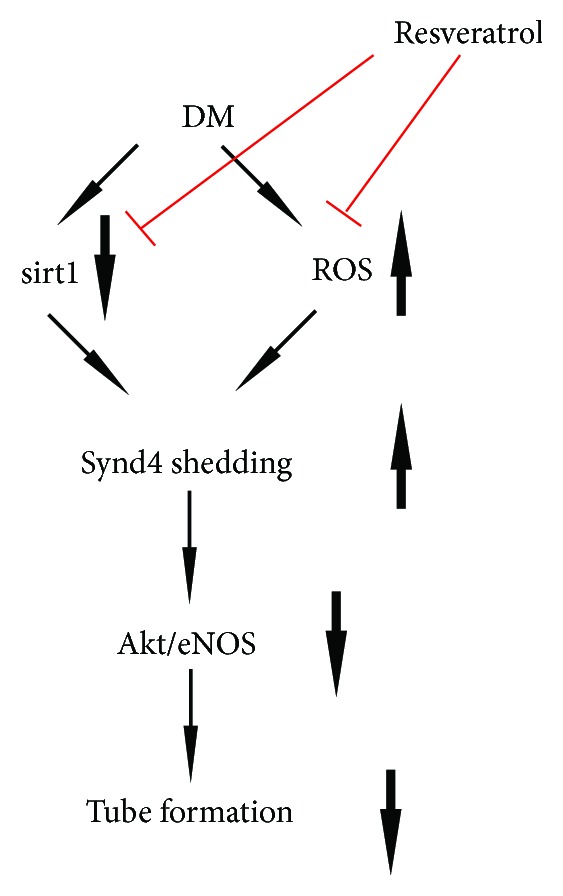
Schematic diagram for the possible mechanisms of the protective effect of resveratrol in AGE-induced EPCs. In the diabetic condition, decreased sirt1 expression and increased ROS production triggers Synd4 shedding, which is responsible for impaired tube formation of EPCs. Treatment with resveratrol improves tube formation of EPCs via the regulation of the sirt1/ROS/Synd4/Akt/eNOS pathway.

Here we showed for the first time that resveratrol normalized AGE-induced impaired tube formation of EPCs via the regulation of the Synd4/Akt/eNOS pathway. Furthermore, resveratrol ameliorated Synd4 shedding in AGE-induced EPCs via the inhibition of oxidative stress (Figure 7).

Synd4 is a transmembrane HSPG that is expressed in many cells including endothelial cells. Synd4 can selectively bind to various matrix components and growth factors, and may facilitate important biological activities [9]. In addition to endothelial cells, Synd4 was mainly expressed in late EPCs in this study as Western blot analysis showed expressions of Synd4 in cell lysate. It was well established that the ectodomain of Synd4 was constitutively shed in various cells as normal turnover, but was accelerated in response to some pathophysiological conditions including diabetes [10, 11, 17]. Importantly, we showed that the ectodomain of Synd4 was significantly downregulated by AGEs in late EPCs, suggesting that Synd4 shedding on late EPCs was an important pathological phenomenon in diabetes.

Oxidative stress has been considered to have a critical role in the development of cardiovascular complications of diabetes, and antioxidative agents have been shown to prevent diabetic complications [18]. Here, it was observed that AGE-induced Synd4 shedding was attenuated by NAC, an antioxidative agent. It suggested that AGEs induced Synd4 shedding in late EPCs via oxidative stress. Furthermore, resveratrol, an antioxidative agent, mimicked the effect of NAC, indicating that resveratrol prevented AGE-induced Synd4 shedding in late EPCs via the inhibition of oxidative stress. Taken together, the data showed that resveratrol reversed AGE-induced Synd4 shedding from late EPCs partly via the inhibition of oxidative stress.

sirt1, a mammalian homologue of sir2, was shown to prevent oxidative stress in diabetes [16, 19]. In the current investigation, we demonstrated that AGE treatment significantly decreased expressions of sirt1 in late EPCs, while resveratrol, an activator of sirt1, normalized the downregulated sirt1 expressions. Recently, we have found that resveratrol treatment inhibited AGE-induced p66shc expressions in late EPCs, but the effect disappeared when AGE-stimulated late EPCs were pretreated with Ex527, an antagonist of sirt1 [16]. Here, the effect of resveratrol on Synd4 shedding was partly reversed by pretreatment with Ex527, suggesting that resveratrol attenuated AGE-induced increased Synd4 shedding in late EPCs via the upregulation of sirt1. Thus, a conclusion is drawn that resveratrol may inhibit Synd4 shedding from late EPCs via the regulation of the sirt1/ROS pathway.

Several studies have demonstrated that Synd4 contributed to endothelial tubulogenesis [12, 20, 21]. Inhibition of Synd4 was shown to attenuate endothelial angiogenesis in HUVECs [20]. Similarly, sustained Synd4 overexpression induced neovascularization in vivo and promoted tube formation of HUVECs in vitro [12, 22]. However, whether Synd4 regulated tube formation of EPCs was not really understood. In the present study, AGEs induced Synd4 shedding as well as impaired tube formation of EPCs, however, overexpression of Synd4 reversed the dysfunction of EPCs induced by AGEs, suggesting that Synd4 was partly involved in the angiogenesis of EPCs. Recently, we have found that AGEs impaired EPC migration via Synd4 shedding [10]. Taken together, these data lent additional evidence to the hypothesis that diabetes impaired angiogenesis via the induction of Synd4 shedding from late EPCs.

It was shown that Synd4 regulated Akt activation via the recruitment of the mTOR complex 2 (mTORC2) and PDK1/PAK signaling pathway [23, 24]. eNOS was shown to be a critical player in angiogenesis and was usually considered as a downstream protein of Akt. Thus, we detected eNOS activation in this investigation. In line with a previous study suggesting increased phosphorylation of eNOS in HUVECs overexpressing Synd4 [12], we observed that overexpression of Synd4 in EPCs induced activation of the Akt/eNOS pathway. It suggested that Synd4 might promote tube formation of EPCs via the Akt/eNOS pathway.

We can conclude that AGE-induced Synd4 shedding via oxidative stress accelerates impaired tube formation of late EPCs in diabetes, and resveratrol improves tube formation of late EPCs via the regulation of Synd4 shedding and the Akt/eNOS pathway. Therefore, it may represent a novel mechanism by which resveratrol prevents diabetes-induced endothelial dysfunction. However, further progress has been mired by unresolved questions around Synd4 shedding induced by oxidative stress, and in vitro studies are warranted to determine the effect of resveratrol in Synd4 shedding and neovascularization in diabetes.

## Data Availability

The data used to support the findings of this study are available from the corresponding author upon request.
